# Effects of metformin and donepezil on the prevention of doxorubicin-induced cardiotoxicity in breast cancer: a randomized controlled trial

**DOI:** 10.1038/s41598-023-40061-4

**Published:** 2023-08-07

**Authors:** Nichanan Osataphan, Arintaya Phrommintikul, Krit Leemasawat, Areewan Somwangprasert, Nattayaporn Apaijai, Supanai Suksai, Wachiranun Sirikul, Siriluck Gunaparn, Siriporn C. Chattipakorn, Nipon Chattipakorn

**Affiliations:** 1 Division of Cardiology, Department of Internal Medicine, Faculty of Medicine, Chiang Mai University, Chiang Mai, Thailand; 2 Cardiac Electrophysiology Research and Training Center, Faculty of Medicine, Chiang Mai University, Chiang Mai, 50200 Thailand; 3 Center of Excellence in Cardiac Electrophysiology Research, Chiang Mai University, Chiang Mai, Thailand; 4 Department of Surgery, Faculty of Medicine, Chiang Mai University, Chiang Mai, Thailand; 5 Department of Community Medicine, Faculty of Medicine, Chiang Mai University, Chiang Mai, Thailand; 6 Department of Oral Biology and Diagnostic Sciences, Faculty of Dentistry, Chiang Mai University, Chiang Mai, Thailand; 7 Cardiac Electrophysiology Unit, Department of Physiology, Faculty of Medicine, Chiang Mai University, Chiang Mai, Thailand

**Keywords:** Cancer, Cardiology

## Abstract

Doxorubicin (DOX) causes deleterious cardiotoxicity. We aimed to investigate the protective roles of metformin and donepezil against DOX-induced cardiotoxicity. In this randomized-controlled trial, 143 female breast cancer patients were enrolled. Metformin (n = 43), donepezil (n = 52), or placebo (n = 48) were prescribed during DOX treatment. The primary endpoint was a proportion of patients with high sensitivity troponin-I (hsTnI) more than the 99th percentile value (> 15.6 ng/L) after DOX treatment. The secondary outcomes were the changes in the hsTnI, N-terminal pro-B-type natriuretic peptide (NT-proBNP), left ventricular ejection fraction (LVEF), global longitudinal strain (GLS) and peripheral blood mononuclear cells analysis for mitochondrial respiration. Baseline characteristics were similar between the groups. The primary endpoint occurred in 58.54% of metformin group, 76.92% in donepezil group, and 69.77% in placebo group (p = 0.215). The level of hsTnI increased after receiving DOX with subsequent decline in LVEF and GLS. Metformin and donepezil did not attenuate hsTnI elevation, LVEF or GLS reduction. There was no significant change in NT-proBNP level. Mitochondrial respiratory dysfunction was observed in the placebo and donepezil groups. However, metformin preserved mitochondrial respiration during DOX therapy. In conclusion, co-treatment with metformin or donepezil did not prevent myocardial injury. Metformin had a favorable mitochondrial outcome and warranted future studies.

## Introduction

Cardiovascular diseases and cancer are among the primary causes of global mortality. Advances in cancer treatment have increased cancer patients' survival rates. However, a significant number of patients develop cardiotoxicity related to cancer treatment, and this condition is associated with a poor prognosis^1^. Doxorubicin is an effective chemotherapeutic drug for treatment of various solid and hematologic malignancies. Although it has high efficacy in cancer treatment, doxorubicin can cause left ventricular systolic dysfunction and heart failure in a dose-dependent manner^2^. Several mechanisms have been proposed including mitochondrial dysfunction, oxidative stress leading to reactive oxygen species production and dysregulation of autophagy^3^. The current European Society of Cardiology (ESC) guideline suggests initiating ACEI or ARB and beta-blockers as a primary prevention for high-risk patients receiving doxorubicin^2^. However, the benefit of these cardioprotective agents was modest and had only minimal impact on clinical outcomes^2^.

Metformin is an anti-diabetic agent which has long been use in the treatment of type 2 diabetes mellitus. Evidence from in vitro studies and animal models indicated that metformin could protect against doxorubicin-induced cardiotoxicity^4–8^. The proposed mechanisms are through its antioxidant effects, inhibition of apoptotic pathway and preservation of mitochondrial function^6,7^. Additionally, metformin could preserve cellular energy in doxorubicin model and exerts cardioprotective effects by activation of adenosine monophosphate-activated protein kinase (AMPK) pathway^5,8^.

Donepezil, an acetylcholinesterase inhibitor, is used primarily in the treatment of dementia and Alzheimer’s disease. Interestingly, an observational study showed that patients treated with donepezil had a lower risk of cardiovascular mortality^9^. This effect could be due to the stimulation of parasympathetic activity by increased plasma level of acetylcholine. In an *in-vivo* model of doxorubicin-induced cardiotoxicity, pretreatment with donepezil attenuated mitochondrial dysfunction and apoptosis in doxorubicin-treated rats^10^.

To date, there is no clinical study investigating the role of metformin or donepezil in the prevention of doxorubicin-induced cardiotoxicity. We hypothesized that administration of metformin or donepezil prior to treatment of doxorubicin prevents doxorubicin-induced cardiotoxicity in breast cancer patients.

## Methods

### Study design

This study was a randomized, double blind, double dummy, placebo-controlled trial conducted at Division of Cardiology, Chiang Mai University hospital, Thailand. Breast cancer patients scheduled to receive doxorubicin were referred from primary physician to determine their eligibility to the trial. The study protocol was approved by the Ethics Committee of Faculty of Medicine, Chiang Mai University (Study Code: MED-2563-07001) and registered at Thai Clinical Trials Registry (TCTR), identification number TCTR20200116007, first trial registration 16/01/2020. The study complied with the Declaration of Helsinki. Written informed consent was obtained from each patient.

### Study patients

Patients were eligible if they were female aged ≥ 18 years and diagnosed with breast cancers with an indication to receive adjuvant or neoadjuvant chemotherapy with doxorubicin. The chemotherapy regimen comprises of four cycles of doxorubicin 60 mg/m^2^ and cyclophosphamide 600 mg/m^2^ every 21 days (4AC regimen; doxorubicin cumulative dose 240 mg/m^2^) or six cycles of 5-fluoruoracil 500 mg/m^2^ plus doxorubicin 50 mg/m^2^ and cyclophosphamide 1000 mg/m^2^ every 21 days (6FAC regimen; cumulative dose of doxorubicin 300 mg/m^2^). Exclusion criteria were metastatic breast cancer at diagnosis (except lymph node and bone metastasis), other active malignancies, prior history of chemotherapy or chest wall radiation, prior cardiovascular disease with LVEF < 53% or severe valvular heart disease, estimated glomerular filtration rate (eGFR) < 30 ml/min/1.73 m^2^, corrected QT interval ≥ 500 ms from baseline electrocardiogram (ECG), patients with diabetes mellitus or currently receiving metformin or donepezil, allergy or contraindication to metformin or donepezil, life expectancy < 1 year and pregnancy or lactating.

### Randomization, allocation, and intervention

Patients were randomized on a 1:1:1 ratio to receive metformin, donepezil or placebo using a web-based platform with blinded allocation. The randomization included a pre-stratified according to adjuvant or neoadjuvant status as surgery may affect cardiac troponin level. Randomization was performed by using a permuted block design. The randomization codes and allocation groups were secured at the research unit at the division of cardiology independent of the trial investigators.

Metformin, donepezil and placebo were commenced one day prior to chemotherapy. The intervention drugs were administered in a double-dummy technique. In metformin group, we decided to use extended-release metformin to attenuate gastrointestinal side effects. Metformin was initiated as 1000 mg daily (1 tablet) and titrated to 2000 mg (2 tablets once daily) after 1 week, if well-tolerated. Co-administration of sham-donepezil was started at half tablet and increased to one tablet after 1 week. In donepezil group, donepezil was started at 5 mg (half tablet) and increased to 10 mg (1 tablet) after 1 week, if well-tolerated. Co-treatment with sham-metformin was initiated one tablet then increased to two tablets after 1 week. The placebo group was treated with sham-metformin and sham-donepezil with the same titration protocol as in the intervention groups. All drugs were continued until completion of doxorubicin therapy.

### Study procedures: blood sample collection

**Figure 1:**
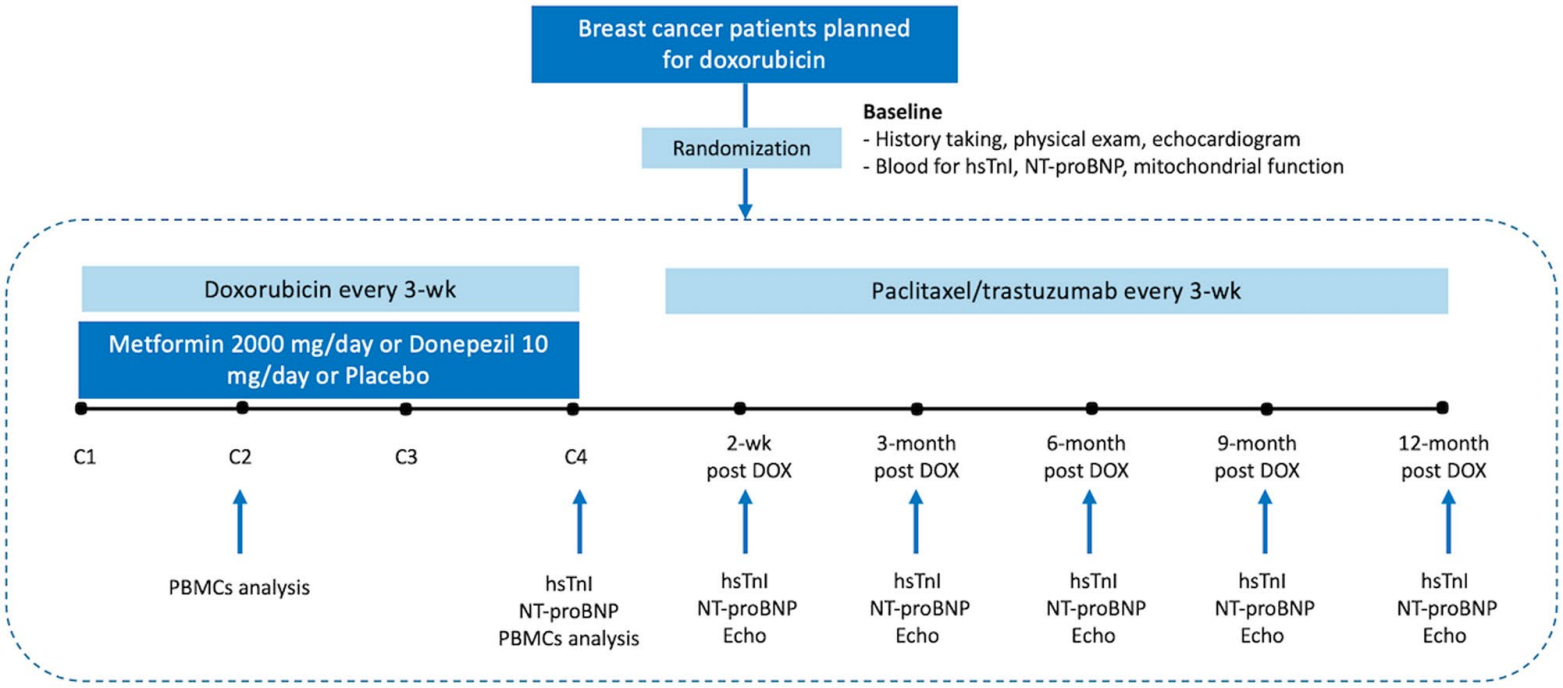
Trial protocol. Baseline evaluation included clinical data collection, echocardiogram, and blood test for biomarkers and PBMCs analysis. Blood samples were obtained at baseline, at 2nd cycle, 4th cycle, 2-week post-DOX and every 3-month until 12-month post-DOX. Echocardiogram was repeated at 2-week post-DOX and every 3-month until 12-month post-DOX. *PBMCs* peripheral blood mononuclear cells, *DOX* doxorubicin, *C* cycle.

For patients in the adjuvant group, clinical data were collected at baseline after surgery and before initiation of chemotherapy. Patients in the neoadjuvant group were assessed for baseline data before initiation of chemotherapy and before surgery. Blood sample collection included an assessment of high sensitivity troponin-I (hsTnI), N-terminal pro B-type natriuretic peptide (NT-proBNP) and peripheral blood mononuclear cells (PBMCs) analysis for mitochondrial respiration and cell death. Echocardiogram was performed to assess baseline cardiac function. Blood for PBMCs analysis was repeated at 2nd cycle, 4th cycle, and 6th cycle (for 6FAC regimen) immediately after complete infusion of chemotherapy in each cycle. Cardiac biomarkers were assessed at 4th cycle, 6th cycle (for 6FAC regimen) immediately after complete infusion of chemotherapy in each cycle and were repeated at 2-week and 3-month, 6-month, 9-month and 12-month after completion of doxorubicin therapy. Highest level of hsTnI between the final cycle and at 2-week after doxorubicin was used for primary outcome analysis. Patients were re-evaluated with echocardiogram at 2 weeks, 3-month, 6-month, 9-month and 12-month after completion of doxorubicin therapy. Patients implanted with breast silicone were excluded from echocardiographic analysis. After completion of doxorubicin treatment, some patients may need further treatment with paclitaxel or trastuzumab, which depended on their tumor status. However, the primary outcome of hsTnI level was measured prior to the administration of paclitaxel and trastuzumab. The study protocols are illustrated in Fig. 1.

### Study procedures: cardiac biomarkers

Troponin-I was determined using the Abbott/Architect stat hsTnI assay ARCHITECT i2000SR Diagnostic System (Abbott) and measured by chemiluminescent microparticle immunoassay (CMIA). The limit of detection ranged from 1.1 to 1.9 ng/L, and the coefficients of variation below 10% was 4.7 ng/L. For patients with hsTnI level below 2.0 ng/L, we defined the level as 1.9 ng/L and used this number for analysis. The gender-specific 99th percentile for female was 15.6 ng/L. NT-proBNP was evaluated with electrochemiluminescence immunoassay by using the Cobas e601 system (Roche Diagnostics). The analytical measurement range of NT-proBNP was 5–35,000 pg/mL. The NT-proBNP level more than 125 ng/L indicated an elevation in cardiac filling pressure^11^.

### Study procedures: PBMCs isolation and mitochondrial respiration

For mitochondrial function, twenty mL of blood was collected from all participants. In order to isolate PBMCs, a Ficoll density gradient centrifugation was used. Measurements of cellular oxidative stress, mitochondrial oxidative stress, and mitochondrial mass were performed using PBMCs. In a brief, after the initial centrifugation (1000*g* for 10 min), white and red blood cells were retrieved, and then re-suspended in phosphate buffer saline solution (PBS). Then, white and red blood cells were diluted and overlaid on Ficoll-Paque reagent (Histopaque, Sigma-Aldrich, MI, USA), and centrifuged at 400*g* for 30 min. The ring of PBMCs at the Ficoll/plasma interface was retrieved after centrifugation and washed twice with 10 ml of PBS. Hemocytometer method was applied to count the number of PBMCs following the final centrifugation. PBMCs were used to measure oxygen consumption during Mito Stress test application by a high throughput Agilent Seahorse XFe96 system (Agilent technologies, CA, USA). PBMCs were loaded into 96 well plate with base medium, and basal respiration was determined. The complex V inhibitor (Oligomycin) was added, and ATP production was determined. Then, the potent uncoupler of oxidative phosphorylation (FCCP) was added, maximal respiration and spare respiratory capacity were determined.

### Study procedure: cell death determination in PBMCs

2 × 10^5^ cells of PBMCs were used, and cells were stained with FITC Annexin V apoptosis detection kit (BD biosciences, New Jersey, USA). FITC Annexin V was used to quantitatively determine the percentage of cells within a population that were actively undergoing apoptosis. Flow cytometric analysis by propidium iodide was used to distinguish viable from nonviable cells, the percentage of viable cells were calculated.

### Study procedures: echocardiogram

Transthoracic echocardiography was performed using the IE33 Philips machine (Koninklijke Philips N.V., Netherlands) with 2D image acquisition with the Philips S5-1 transducer and harmonic imaging. Complete 2D transthoracic echocardiography was performed according to the recommendations of the American Society of Echocardiography (ASE)^12^. Left ventricular ejection fraction (LVEF) was measured by modified biplane Simpson’s technique. Global longitudinal strain (GLS) was performed by using AutoSTRAIN (TOMTEC, Germany). Cases with more than two inadequately tracked segments were excluded from strain analysis. All images were stored in system then were analyzed by two independent cardiologists who were blinded to the allocation groups. Variation of more than 10% in the measurement required a reassessment by the third cardiologist.

### Study endpoints

The primary endpoint was a proportion of patients with hsTnI level more than the cut-off value (> 15.6 ng/L) at the final cycle or 2-week after doxorubicin treatment. Secondary endpoints were changes in hsTnI level, NT-proBNP level, mitochondrial function, LVEF and GLS after doxorubicin treatment. We used troponin-I as the primary outcome since it exhibits a stronger correlation with cardiotoxicity compared to NT-proBNP^13^ and can earlier detect cardiotoxicity compared to LVEF^2^. In patients who developed low LVEF or GLS reduction during treatment, causes of left ventricular systolic dysfunction were investigated according to standard of care^2^. Clinical endpoints including the incidence of chemotherapy-related cardiac dysfunction (CTRCD) and all-cause death were collected. CTRCD was defined as an LVEF reduction of more than 10% to the value below 53%^12^. If patients developed symptom of heart failure, clinical assessment and further investigations were conducted. Standard treatments for heart failure were administered to patients with preserved or reduced ejection fraction. This study reported the secondary outcomes and clinical endpoints followed to 3-month after doxorubicin therapy.

### Statistical analysis

The categorical data were presented as a frequency (percentage) and compared between the study groups using Fisher’s exact test. For parametric data, the continuous data were reported as a mean with standard deviation (SD), and for non-parametric data, as a median with interquartile range (IQR). The multiple comparisons between the intervention groups and the placebo were performed using one-way ANOVA test for parametric data or Kruskal–Wallis test for non-parametric data. If there is a significant difference from the aforementioned tests, a post-hoc pairwise comparison using Bonferroni correction was performed to identify a significantly different pair. The analysis of the primary outcome was performed in accordance with a modified intention-to-treat principle, which included all patients who had undergone randomization and had available primary outcome data. The repeated measurement outcomes of hsTnI, NT-proBNP, LVEF, and GLS were analyzed using a multilevel mixed-effects linear regression with time interaction to determine the effect of treatments or chemotherapy on clinical outcomes over time. Based on the anticipated change in hsTnI and NT-proBNP levels following chemotherapy treatment, a quadratic time interaction term was included in the models. The changes in mitochondrial function in each group and the comparison between the study groups in each period were analyzed by Kruskal–Wallis test with post-hoc pairwise comparisons using Bonferroni correction. Two-tailed p-values of 0.05 were considered statistically significant. All statistical analysis was performed by the statistical software STATA16 (Stata Corp. 2019, Stata Statistical Software: Release 16, Stata Corp. LLC, College Station, TX, USA).

### Sample size calculation

The primary endpoint of this study was the proportion of patients with elevated hsTnI more than the cut-off value (hsTnI > 15.6 ng/L) at the final cycle or 2-week after doxorubicin treatment. From previous study, the incidence of hsTnI elevation post doxorubicin therapy was 41.6%^14^. We assumed a 40% reduction in the incidence of hsTnI elevation with the addition of intervention drugs. With estimation of a loss follow up of 5%, alpha level of 0.05 and 80% power, a total of 132 patients in each arm was required. Therefore, the planned recruitment of patients in this study was 396.

### Ethical approval

Effects of metformin and donepezil on the prevention of doxorubicin-induced cardiotoxicity in breast cancer patients: a randomized controlled trial was approved by the ethics committee of the Faculty of Medicine, Chiang Mai University, approval number 111/2563. The investigations were carried out in accordance with the Declaration of Helsinki, including the written informed consent of all participants.

## Results

### Patients

**Figure 2:**
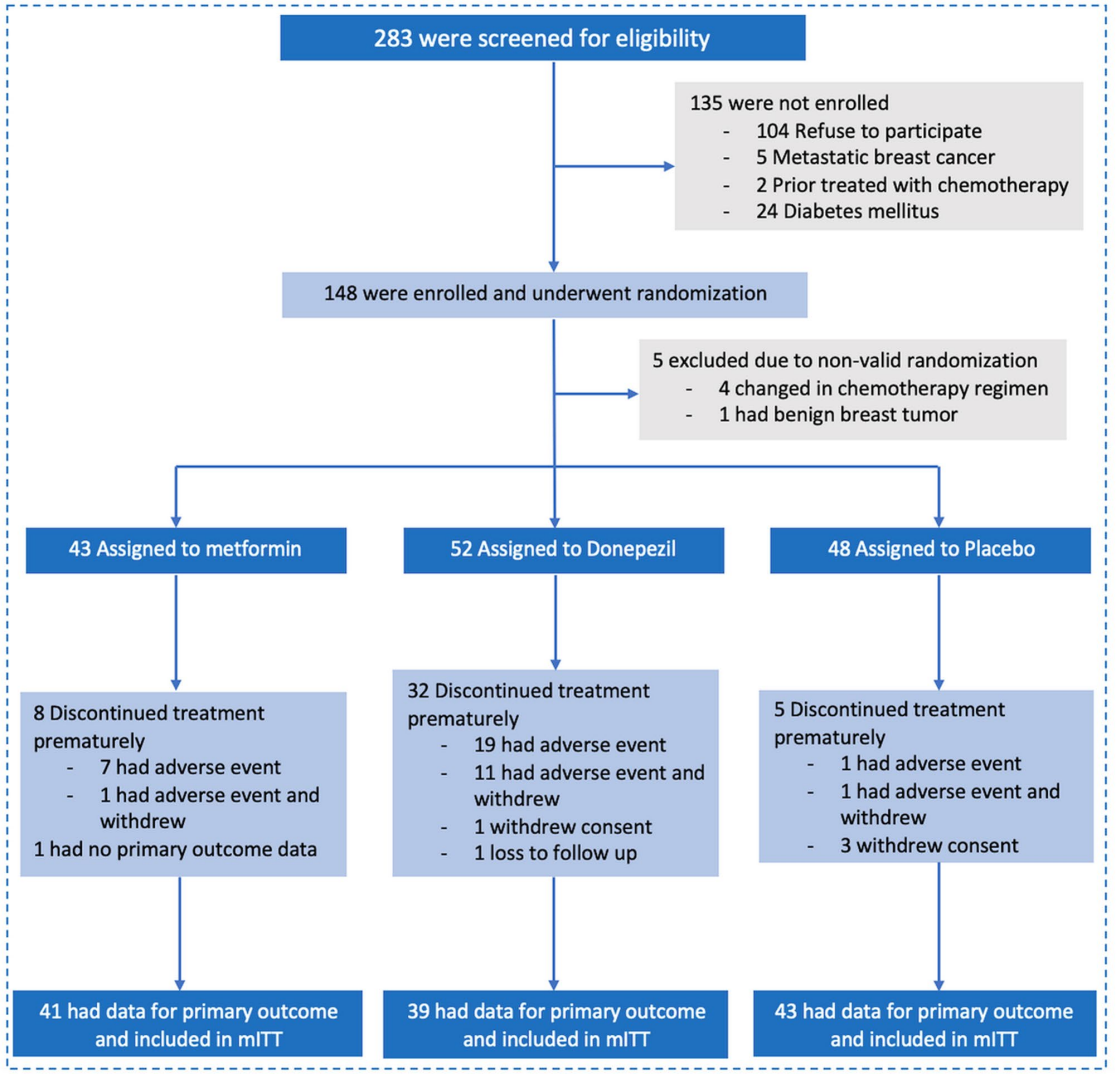
Consort diagram. Patients were screened for eligibility then were randomized to metformin, donepezil, or placebo group. Patients who had primary outcome data were included in the modified intention-to-treat analysis. *mITT* modified intention-to-treat.

From June 2020 to May 2022, a total of 283 breast cancer patients were screened for eligibility. The investigators decided to stop the trial before reaching the intended sample size due to the slower than expected enrollment attributable to the pandemic of coronavirus disease 2019 (Covid-19). A total of 148 patients underwent randomization to the groups of metformin, donepezil, or placebo. Five patients were excluded due to non-valid randomization. Of the remaining 143 patients, 43 were in metformin group, 52 were in donepezil group and 48 were in placebo group. There were 123 patients who had data for primary outcome analysis. The study consort diagram is presented in Fig. 2.

Baseline characteristics are presented in Table 1. Patients in the metformin group was significantly younger than patients in the donepezil and placebo group (48.0 ± 10.43 vs 55.13 ± 10.27 vs 55.88 ± 10.73 years; p < 0.001, respectively). The prevalence of hypertension was low with non-significant difference between the groups. None of the patients in this study had previous history of coronary artery disease or myocardial infarction. Most patients underwent breast surgery before chemotherapy (adjuvant). All of the patients completed chemotherapy course comprised of four cycles of doxorubicin (4AC regimen) and six cycles of doxorubicin (6FAC regimen) in 137 patients (95.8%) and 6 patients (4.2%) respectively. Forty-one patients had HER2-postive breast cancer tumor status and received trastuzumab treatment after doxorubicin. The cumulative dose of doxorubicin was similar in all groups (metformin 229.38 ± 26.14 vs donepezil 221.97 ± 10.48 and placebo 228.48 ± 33.42 mg; p = 0.28). Systolic blood pressure, diastolic blood pressure and heart rate were comparable between the groups. The mean baseline LVEF was approximately 69% in all groups (p = 0.98). The levels of baseline hsTnI and NT-proBNP were not different between the groups (Table 1).

**Table 1:** Baseline characteristics.

	Metformin (n = 43)	Donepezil (n = 52)	Placebo (n = 48)	p-value
Age, years	48.0 ± 10.43	55.13 ± 10.27	55.88 ± 10.73	< 0.001
Clinical history				
Hypertension	11 (25.58)	12 (23.08)	9 (18.75)	0.73
Anti-hypertensive agents				
ACEI	3 (6.98)	3 (5.77)	2 (4.17)	0.84
ARB	2 (4.65)	3 (5.77)	3 (6.25)	0.94
Beta-blocker	0	0	0	NA
CCB	4 (9.30)	8 (15.38)	5 (10.42)	0.61
Dyslipidemia	7 (16.28)	11 (21.15)	9 (18.75)	0.83
Atrial fibrillation	0	0	1 (1.92)	0.41
Breast cancer data				
Breast cancer therapy				
Adjuvant	30 (69.77)	33 (63.46)	34 (70.83)	0.70
Neoadjuvant	13 (30.23)	19 (36.54)	14 (29.17)	
HER-2 positive	14 (32.56)	16 (30.77)	11 (22.92)	0.55
Doxorubicin cumulative dose, mg/m^2^	229.38 ± 26.14	221.97 ± 10.48	228.48 ± 33.42	0.28
Clinical parameters				
Systolic blood pressure, mmHg	128.67 ± 16.30	130.71 ± 18.24	128.19 ± 18.47	0.75
Diastolic blood pressure, mmHg	73.19 ± 10.16	74.57 ± 9.69	74.66 ± 10.77	0.74
Heart rate, beat/min	85.19 ± 11.0	83.81 ± 13.45	86.87 ± 12.91	0.48
Left ventricular ejection fraction, %	69.60 ± 4.6	69.80 ± 5.06	69.81 ± 6.08	0.98
Serum hsTnI, ng/L	1.9 (1.9–1.9)	1.9 (1.9–1.9)	1.9 (1.9–1.9)	0.47
Serum NT-proBNP, ng/L	31.76 (23.5–52.2)	47.84 (27.6–68.6)	41.55 (28.3–74.32)	0.17

Data presented as n (%), mean ± standard deviation or median and interquartile range.

*ACEI* angiotensin-converting enzyme inhibitor, *ARB* angiotensin receptor blocker, *CCB* calcium channel blocker, *hsTnI* high sensitivity troponin-I, *NT-proBNP* N-terminal pro B-type natriuretic peptide.

### Trial treatments and follow-up

The trial drugs were discontinued prematurely in 8 patients (18.6%) in the metformin group, 32 patients (61.5%) in the donepezil group and 5 patients (10.4%) in the placebo group. The main reason for treatment withdrawal was related to drug adverse events. Pill count adherence showed at least 80% compliance in 66.7% of the placebo group, 58.1% for the metformin group and 32.7% in the donepezil group. Six patients (13.9%) in the metformin group and three patients (5.8%) in the donepezil group took half of the prescribed dose of metformin (1000 mg) and donepezil (5 mg). Only one patient (donepezil group) was loss to follow-up.

### Primary outcome

**Figure 3:**
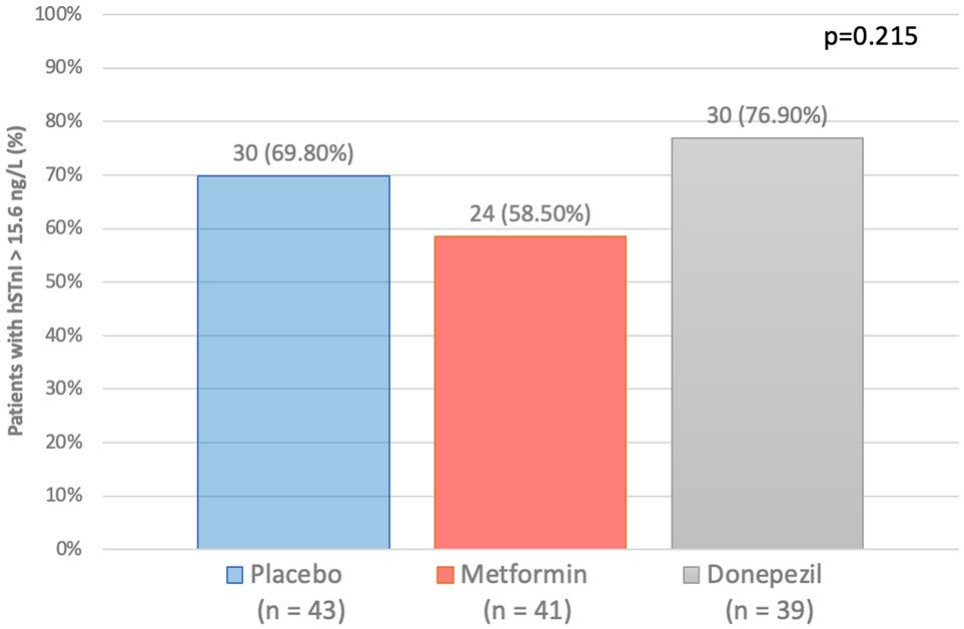
Bar graph of primary outcome. Proportion of patients with hsTnI more than the 99th percentile cut-off value (> 15.6 ng/L) was presented. There was no significant different in the proportion of patients with hsTnI more than the cut-off value between placebo, metformin, and donepezil. *hsTnI* high sensitivity troponin-I.

The primary outcome was a proportion of patients with the maximal hsTnI level more than 99th percentile value (> 15.6 ng/L) at the final cycle or 2-week post doxorubicin treatment. The primary outcome occurred in 24 (58.54%) patients in the metformin group, 30 (76.92%) in the donepezil group, and 30 (69.77%) in the placebo group (p-value = 0.215). These results are presented in Fig. 3.

### Secondary outcomes: hsTnI and NT-proBNP

**Figure 4:**
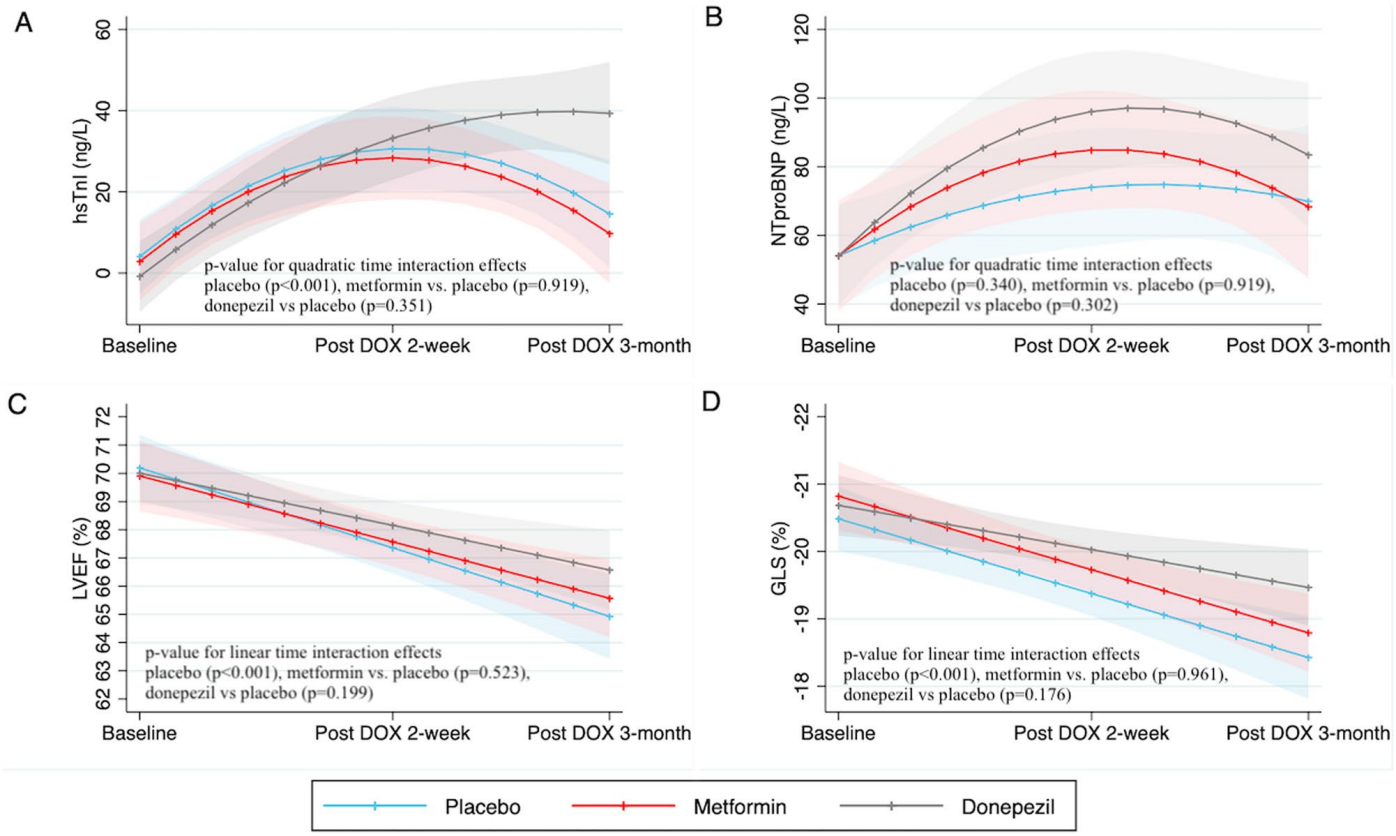
Predictive margins of hsTnI, NT-proBNP, LVEF, and GLS during the follow-up period between metformin, donepezil, and placebo. (**A**) hsTnI; Treatment with doxorubicin led to an increase in hsTnI level at 2-week with a gradual reduction at 3-month (p-value for quadratic time interaction effects < 0.001 in placebo arm). The rate of hsTnI changes were not different in patients taking metformin (p = 0.919) and donepezil (p = 0.351) when compared to placebo. (**B**) NT-proBNP; There was no significant change in the level of NT-proBNP during treatment with doxorubicin (p-value for quadratic time interaction effects = 0.340 in placebo arm). When compared to placebo, taking metformin and donepezil had similar changes in NT-proBNP level (metformin; p = 0.919 and donepezil; p = 0.302). (**C**) LVEF; There was a gradual reduction in LVEF overtime (p-value for linear time interaction effects < 0.001 in placebo arm). The rate of LVEF reduction was not statistically difference in patients taking metformin (p = 0.523) and donepezil (p = 0.199) when compared to placebo. (**D**) GLS; The level of GLS decreased after doxorubicin (p-value for linear time interaction effects < 0.001 in placebo arm). When compared to placebo, there was no significant difference in the rate of GLS reduction in patients taking metformin (p = 0.961) and donepezil (p = 0.176). *hsTnI* high sensitivity troponin-I, *NT-proBNP* N-terminal pro B-type natriuretic peptide, *LVEF* left ventricular ejection fraction, *GLS* global longitudinal strain, *DOX* doxorubicin.

Cardiac biomarker results are shown in Table 2 and Fig. 4. Regression analysis with either linear or quadratic time effects showed that treatment with doxorubicin led to a significant increase in hsTnI at 2-week after doxorubicin treatment with gradual decline over time. However, co-treatment with metformin or donepezil did not attenuate the increase in hsTnI level. In addition, treatment with doxorubicin did not significantly increase the level of NT-proBNP. There was no significant change of NT-proBNP level in patients treated with either metformin or donepezil. Patients whose baseline hsTnI or NT-proBNP were higher had greater increase in their levels after receiving doxorubicin.

**Table 2:** Regression analysis of hsTnI and NT-proBNP level.

Parameters	hsTnI			NT-proBNP		
	β co-efficient	95% CI	*p-*value	β co-efficient	95% CI	*p-*value
Baseline	6.12	4.14 to 8.10	** < 0.001**	1.00	0.88 to 1.11	** < 0.001**
Fixed effects						
Metformin	− 1.25	− 14.43 to 11.93	0.853	− 0.01	− 22.23 to 22.12	0.999
Donepezil	− 4.90	− 17.50 to 7.70	0.446	− 0.01	− 20.85 to 20.87	1.000
Linear interaction effects						
Linear time effect (placebo)	3.64	1.74 to 5.53	** < 0.001**	2.34	− 0.95 to 5.62	0.163
Metformin × time	0.01	− 2.68 to 2.70	0.997	1.79	− 2.91 to 6.50	0.455
Donepezil × time	− 0.17	− 2.83 to 2.49	0.900	2.92	− 1.71 to 7.55	0.216
Quadratic interaction effects						
Quadratic time effect (placebo)	− 0.12	− 0.20 to − 0.05	**0.001**	− 0.06	− 0.20 to − 0.07	0.340
Metformin × time^2^	− 0.01	− 0.11 to 0.10	0.919	− 0.07	− 0.26 to 0.11	0.919
Donepezil × time^2^	0.05	− 0.06 to 0.16	0.351	− 0.09	− 0.28 to 0.09	0.302
Constant	− 10.40	− 20.29 to − 0.51	0.039	0.07	− 16.37 to 16.51	0.993

*hsTnI* high sensitivity troponin-I, *NT-proBNP* N-terminal pro B-type natriuretic peptide.

Significant values are given in bold.

### Secondary outcomes: LVEF and GLS

The results of LVEF and GLS are presented in Table 3 and Fig. 4. Treatment with doxorubicin resulted in a significant reduction in LVEF and GLS over time. At two weeks and 3-month after doxorubicin treatment, the decline in the LVEF and GLS in patients receiving either metformin or donepezil was comparable to that observed in the placebo group, indicating that those interventions did not attenuate the LVEF and GLS reduction. Patients with lower baseline LVEF or GLS experienced greater post-doxorubicin reduction in LVEF and GLS.

**Table 3:** Regression analysis of LVEF and GLS.

Parameters	LVEF (%)			GLS (%)		
	β co-efficient	95% CI	*p-*value	β co-efficient	95% CI	*p-*value
Baseline	0.71	0.62 to 0.80	** < 0.001**	0.70	0.62 to 0.76	** < 0.001**
Fixed effects						
Metformin	− 0.29	− 2.00 to 1.42	0.742	− 0.33	− 1.04 to 0.36	0.345
Donepezil	− 0.19	− 1.78 to 1.40	0.815	− 0.20	− 0.85 to 0.46	0.552
Linear interaction effects						
Linear time effect (placebo)	− 0.20	− 0.28 to − 0.12	** < 0.001**	0.08	0.05 to 0.11	** < 0.001**
Metformin × time	0.35	− 0.07 to 0.15	0.523	− 0.01	− 0.05 to 0.04	0.961
Donepezil × time	0.07	− 0.03 to 1.78	0.199	− 0.03	− 0.08 to 0.14	0.176
Constant	20.27	13.72 to 26.83	** < 0.001**	− 6.11	− 7.59 to − 4.63	** < 0.001**

*LVEF* left ventricular ejection fraction, *GLS* global longitudinal strain.

Significant values are given in bold.

### Secondary outcomes: mitochondrial respiration and cell death in PBMCs

**Figure 5:**
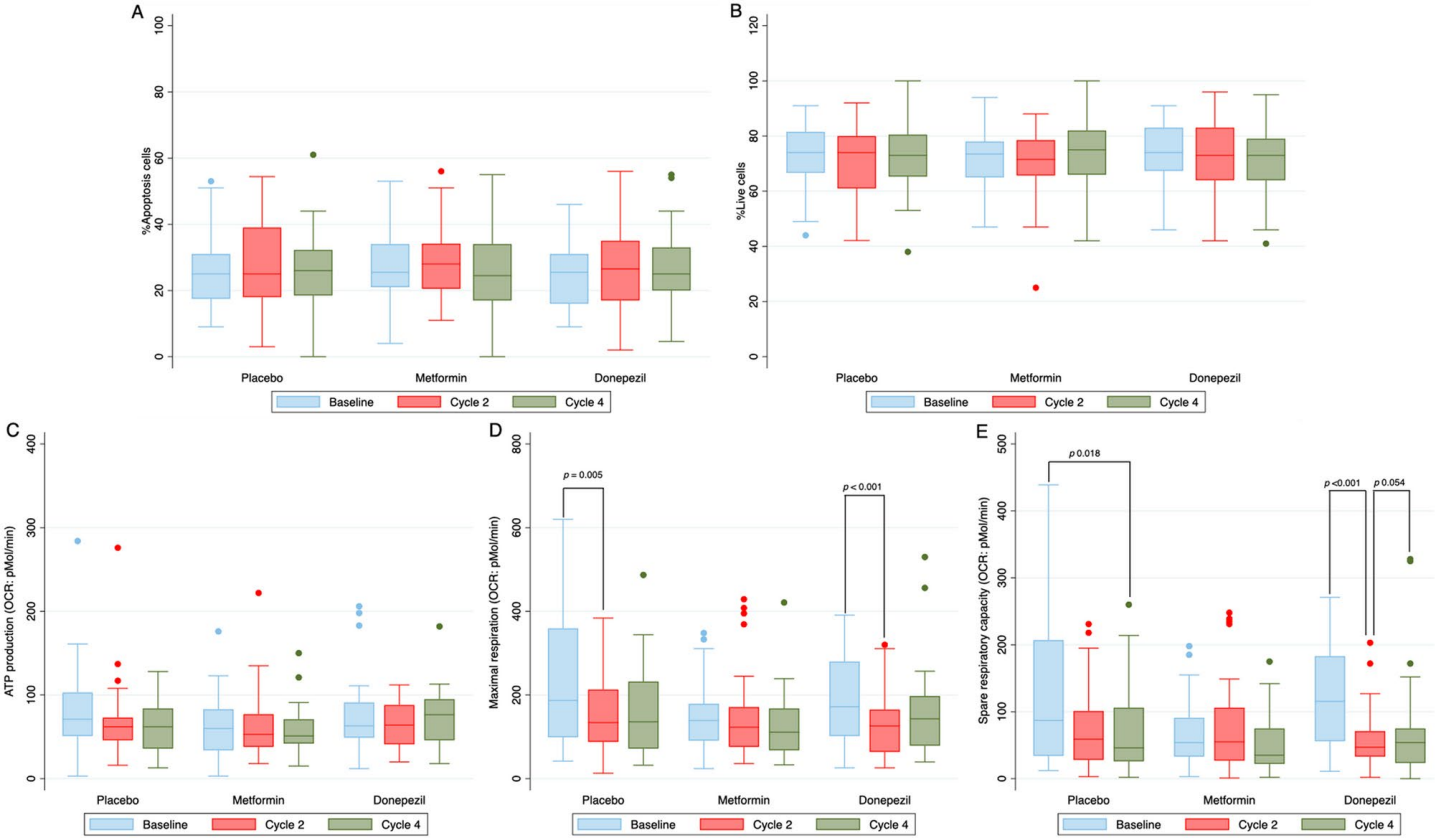
PBMCs analysis of apoptotic cells, live cells and mitochondrial respiration at 2nd cycle and 4th cycle of doxorubicin between metformin, donepezil, and placebo. (**A**) Number of apoptotic cells. (**B**) Number of live cells. (**C**) Level of ATP. (**D**) Level of maximal mitochondrial respiration. (**E**) Level of spare respiratory capacity. After DOX treatment, the number of apoptotic cells and live cells were comparable across all groups. ATP levels were not different across all groups. There was a significant reduction in maximal mitochondrial respiration and the spare respiratory capacity level during doxorubicin treatment in placebo and donepezil groups. Metformin preserved maximal mitochondrial respiration and the spare respiratory during doxorubicin treatment. *PBMCs* peripheral blood mononuclear cells, *DOX* doxorubicin, *ATP* adenosine triphosphate.

From the analysis of PBMCs, the number of apoptotic cells and live cells remain constant after treatment with doxorubicin in all groups (Fig. 5). In the placebo and donepezil groups, there was a significant reduction in maximal mitochondrial respiration at the 2nd cycle. Interestingly, metformin preserved maximal mitochondrial respiration at both 2nd cycle and 4th cycle. For mitochondrial spare respiratory capacity, doxorubicin significantly reduced the spare respiratory capacity level at the 4th cycle as indicated in the placebo group. Donepezil did not attenuate the reduction in mitochondrial spare respiratory capacity. Interestingly, for patients treated with metformin, the spare respiratory capacity was not reduced during treatment with doxorubicin. Adenosine triphosphate (ATP) production was comparable at baseline, 2nd cycle, and 4th cycle in all groups. These results are demonstrated in Fig. 5.

### Clinical endpoints

Only 2 patients met the criteria for CTRCD and both were asymptomatic. Left ventricular dysfunction was detected during the follow-up echocardiogram at 3-month post doxorubicin therapy. Both patients were receiving trastuzumab treatment at the time CTRCD was discovered as they had HER-2 positive status. Cardioprotective treatment with beta-blocker and angiotensin-converting enzyme inhibitor were prescribed. Both patients had an improvement of LVEF to 50% on the follow-up echocardiogram. No patient died during trial follow-up.

### Safety

Adverse events related to intervention drugs are reported in Supplement Table S1. Donepezil was associated with higher palpitation (26.92%; p = 0.003) and nausea/vomiting (48.08%; p < 0.001) compared to the metformin and placebo groups. These side effects resulted in more treatment discontinuation in the donepezil group. When compared to the other two groups, patients receiving metformin had a greater incidence of diarrhea (25.58%; p = 0.03). No patient had hypoglycemia symptoms or urgent visit related to hypoglycemia. There were no significant differences in sleep-related disorders or other cardiovascular symptoms. No patient experienced life-threatening adverse events related to intervention drugs.

## Discussion

To the best of our knowledge, this study is the first RCT to investigate the potential cardioprotective effects of metformin and donepezil in breast cancer patients receiving doxorubicin. Co-treatment with either metformin or donepezil did not reduce the incidence of hsTnI elevation post-doxorubicin therapy. In the placebo group, the level of hsTnI increased significantly after receiving doxorubicin, with consequent decline in LVEF and GLS. However, neither metformin nor donepezil attenuated these effects. The secondary analysis of mitochondrial respiration in PBMCs showed that only metformin could preserve maximal mitochondrial respiration and spare respiratory capacity during doxorubicin treatment.

Previous in vitro and in vivo models suggested that metformin prevented doxorubicin-induced cardiotoxicity^4–8^. Several mechanisms have been discussed, including anti-apoptosis, reducing oxidative stress, and improving mitochondrial function. However, the results from pre-clinical trials did not translate into a favorable clinical outcome in our study. Metformin did not prevent myocardial injury after receiving doxorubicin as compared to placebo. There is only a single in vivo study investigating the role of donepezil in the prevention of doxorubicin-induced cardiotoxicity^10^. Co-treatment with donepezil was shown to reduce cardiac troponin-I, NT-proBNP level and preserved ejection fraction in doxorubicin-treated rats. Decreased mitochondrial damage, apoptosis, and necroptosis were the proposed cardioprotective mechanisms of donepezil. Nonetheless, co-treatment with donepezil in breast cancer patients did not prevent cardiotoxicity in our study. The negative results of both interventional drugs in this clinical study in contrast to the animal models could be explained by several hypothesis. First, animal models might not accurately represent the pathophysiologic diversity of human disease^15^. For instance, cancer might also have an effect on the clinical outcome in our patients, in contrast to the healthy rats used in animal models. Second, poor patients’ compliance, especially in the donepezil group, may have masked the significant benefits of these interventional drugs. Third, the inclusion of patients with low risk of developing CTRCD in our study, who may not benefit from primary prevention with cardioprotective agents. Future study should explore the benefit of these agent in high-risk population.

Secondary analysis of mitochondrial respiration in the PBMCs showed an impaired maximal mitochondrial respiration and spare respiratory capacity in the placebo group. These findings supported the hypothesis that mitochondrial dysfunction is one of the main mechanisms of doxorubicin-induced cardiotoxicity^3^. Interestingly, patients treated with metformin had a preservation of mitochondrial respiration during doxorubicin treatment. It may indicate that metformin had some beneficial effect in the prevention of doxorubicin-induced cardiotoxicity. However, this effect did not translate into clinically significant, suggesting other underlie mechanisms in this context apart from mitochondrial dysfunction. In addition, a relatively short period of prophylaxis treatment with metformin may obscure the protective effects of this drug. A longer duration of metformin co-treatment should be explored in future studies. In contrast to the metformin group, patients treated with donepezil still had an impaired mitochondrial respiration after doxorubicin treatment similar to that seen in the placebo group, suggesting that donepezil could not prevent mitochondrial dysfunction from doxorubicin. Although there was an impairment in mitochondrial respiration during doxorubicin treatment, the number of live cells and apoptotic cells in the PBMCs remained unchanged.

The incidence of troponin-I elevation in our study was higher, compared to previous clinical trials^14,16^. The reason behind this was the use of lower cut-off value of troponin I, as recommended by the assay utilized in our study. The small changes in LVEF in all groups were consistent with several reports^14,17^. As cardiac troponin-I can detect subclinical cardiotoxicity, changes in troponin-I occurred before the reduction in LVEF^2^. There was no significant change of NT-proBNP level after treatment with doxorubicin. Therefore, NT-proBNP level was less correlated with cardiotoxicity, compared to hsTnI. However, patients who had higher baseline hsTnI or NT-proBNP had a greater increased in those levels after doxorubicin treatment, indicating that they are a high-risk population. Long-term monitoring and control of cardiovascular risk factors are encouraged for these individuals. For patients who developed left ventricular dysfunction or clinical heart failure during follow-up, clinical assessment and further investigations were conducted according to the standard of care^2^. Left ventricular systolic dysfunction can be observed due to several causes such as coronary artery disease, stress-induced cardiomyopathy, or chemotherapy. There were only two patients who had left ventricular dysfunction and met the criteria for CTRCD in our study. Based on the clinical assessment, chemotherapy was deemed the most likely cause of their left ventricular dysfunction, as both patients were undergoing trastuzumab treatment when the dysfunction occurred. Both patients received standard heart failure medication, and subsequent follow-up revealed an improvement in LVEF to more than 50%. Overall, the incidence of CTRCD and heart failure in our study were low. The reasons could be that the follow-up period was relatively short, as doxorubicin toxicity can manifest many years after the completion of chemotherapy^18^. Long-term follow-up may show more clinical events in this population.

Our study showed higher discontinuation rates in patients taking donepezil (61.5%) compared to those taking metformin (18.6%) and placebo (10.4%), which was attributed to the adverse drug reactions. The rate of drug withdrawal in the donepezil group in our study was higher, compared to previous clinical trials of donepezil in non-cancer patients^19–21^. The most frequent adverse events of donepezil were palpitation and nausea/vomiting, which were also prevalent among previous clinical trials^20,21^. As cancer patients also experienced nausea and vomiting from chemotherapeutic agents, adding donepezil might worsen these symptoms. Additionally, a very high discontinuation rate suggested that donepezil may not be a suitable interventional drug to be used in this population. Metformin had higher tolerability than donepezil and was comparable to the placebo. Although extended-release metformin was used, diarrhea still occurred more frequently compared to donepezil and placebo. Using metformin in non-diabetic patients did not result in an increased incidence of hypoglycemia, which was consistent with previous study^22^.

### Study limitation

Our study has some limitations that should be addressed. First, because this was a single-center trial, the study population was less heterogeneous. Second, the sample size was lower than expected due to slow enrollment during covid-19, which resulted in the underpowered primary outcome analysis. Third, high discontinuation rate of the investigational drugs and poor compliance may attenuate the efficacy of actual treatment interventions. Fourth, a relatively short follow-up period may result in the lower incidence of the clinical events. Fifth, the use of 2D LVEF and the interobserver variation might affect the accuracy LVEF measurement. However, we have attempted to minimize this issue by the measurement of two independent cardiologists.

### Future trend

Our work provides pertinent information that can be used to guide future research. We have demonstrated that there was mitochondrial dysfunction in PBMCs of patients treated with doxorubicin. The correlation between PBMCs and cardiac function has been evaluated in many recent cardiovascular studies^23–25^. The finding from our study, which demonstrated that metformin preserves mitochondrial respiration in PBMCs, may encourage future research to warrant its potential cardioprotective role in this field. Future studies with larger sample size are needed to confirm this hypothesis.

## Conclusions

In breast cancer patients treated with doxorubicin, co-treatment with either metformin or donepezil did not prevent myocardial injury. Neither metformin nor donepezil attenuated the reduction in LVEF or GLS after doxorubicin treatment. However, at mitochondria level, doxorubicin caused an impaired mitochondrial function. Metformin was the only interventional drug that could preserve mitochondrial respiration during doxorubicin therapy. Future studies should explore possible cardioprotective effects of metformin.

### Supplementary Information

Supplementary Table 1.

## Data Availability

The informed consent given by the participants does not cover data posted in public databases. However, data available should be sent to nipon.chat@cmu.ac.th and the Faculty of Medicine, Chiang Mai University Ethics Committee for approval upon request.

## References

[1] López-Sendón J, Álvarez-Ortega C, Zamora Auñon P (2020). Classification, prevalence, and outcomes of anticancer therapy-induced cardiotoxicity: The CARDIOTOX registry. Eur. Heart J..

[2] Lyon AR, López-Fernández T, Couch LS (2022). 2022 ESC Guidelines on cardio-oncology developed in collaboration with the European Hematology Association (EHA), the European Society for Therapeutic Radiology and Oncology (ESTRO) and the International Cardio-Oncology Society (IC-OS). Eur. Heart J..

[3] Osataphan N, Phrommintikul A, Chattipakorn SC, Chattipakorn N (2020). Effects of doxorubicin-induced cardiotoxicity on cardiac mitochondrial dynamics and mitochondrial function: Insights for future interventions. J. Cell Mol. Med..

[4] Asensio-Lopez MC, Lax A, Pascual-Figal DA, Valdes M, Sanchez-Mas J (2011). Metformin protects against doxorubicin-induced cardiotoxicity: involvement of the adiponectin cardiac system. Free Radic. Biol. Med..

[5] Ashour AE, Sayed-Ahmed MM, Abd-Allah AR (2012). Metformin rescues the myocardium from doxorubicin-induced energy starvation and mitochondrial damage in rats. Oxid. Med. Cell. Long..

[6] Arinno A, Maneechote C, Khuanjing T (2021). Cardioprotective effects of melatonin and metformin against doxorubicin-induced cardiotoxicity in rats are through preserving mitochondrial function and dynamics. Biochem. Pharmacol..

[7] Kelleni MT, Amin EF, Abdelrahman AM (2015). Effect of metformin and sitagliptin on doxorubicin-induced cardiotoxicity in rats: Impact of oxidative stress, inflammation, and apoptosis. J. Toxicol..

[8] Kobashigawa LC, Xu YC, Padbury JF, Tseng Y-T, Yano N (2014). Metformin protects cardiomyocyte from doxorubicin induced cytotoxicity through an AMP-activated protein kinase dependent signaling pathway: An in vitro study. PLoS ONE.

[9] Sato K, Urbano R, Yu C (2010). The effect of donepezil treatment on cardiovascular mortality. Clin. Pharmacol. Ther..

[10] Khuanjing T, Ongnok B, Maneechote C (2021). Acetylcholinesterase inhibitor ameliorates doxorubicin-induced cardiotoxicity through reducing RIP1-mediated necroptosis. Pharmacol. Res..

[11] McDonagh TA, Metra M, Adamo M (2021). 2021 ESC Guidelines for the diagnosis and treatment of acute and chronic heart failure. Eur. Heart J..

[12] Plana JC, Galderisi M, Barac A (2014). Expert consensus for multimodality imaging evaluation of adult patients during and after cancer therapy: A report from the American Society of Echocardiography and the European Association of Cardiovascular Imaging. J. Am. Soc. Echocardiogr..

[13] Michel L, Mincu RI, Mahabadi AA (2020). Troponins and brain natriuretic peptides for the prediction of cardiotoxicity in cancer patients: a meta-analysis. Eur. J. Heart Fail..

[14] Avila MS, Ayub-Ferreira SM, de Barros Wanderley MR (2018). Carvedilol for prevention of chemotherapy-related cardiotoxicity: The CECCY trial. J. Am. Coll. Cardiol..

[15] Mak IW, Evaniew N, Ghert M (2014). Lost in translation: Animal models and clinical trials in cancer treatment. Am. J. Transl. Res..

[16] Cardinale D, Sandri MT, Colombo A (2004). Prognostic value of troponin I in cardiac risk stratification of cancer patients undergoing high-dose chemotherapy. Circulation.

[17] Elitok A, Oz F, Cizgici AY (2014). Effect of carvedilol on silent anthracycline-induced cardiotoxicity assessed by strain imaging: A prospective randomized controlled study with six-month follow-up. Cardiol. J..

[18] Steinherz LJ, Steinherz PG, Tan CTC, Heller G, Murphy ML (1991). Cardiac toxicity 4–20 years after completing anthracycline therapy. JAMA.

[19] Aarsland D, Laake K, Larsen JP, Janvin C (2002). Donepezil for cognitive impairment in Parkinson's disease: A randomised controlled study. J. Neurol. Neurosurg. Psychiatry..

[20] Campbell NL, Perkins AJ, Gao S (2017). Adherence and tolerability of Alzheimer's disease medications: A pragmatic randomized trial. J. Am. Geriatr. Soc..

[21] Hong YJ, Han HJ, Youn YC (2019). Safety and tolerability of donepezil 23 mg with or without intermediate dose titration in patients with Alzheimer's disease taking donepezil 10 mg: A multicenter, randomized, open-label, parallel-design, three-arm, prospective trial. Alzheimers Res. Ther..

[22] Preiss D, Lloyd SM, Ford I (2014). Metformin for non-diabetic patients with coronary heart disease (the CAMERA study): A randomised controlled trial. Lancet Diabetes Endocrinol..

[23] Li P, Wang B, Sun F (2015). Mitochondrial respiratory dysfunctions of blood mononuclear cells link with cardiac disturbance in patients with early-stage heart failure. Sci. Rep..

[24] Shirakawa R, Yokota T, Nakajima T (2019). Mitochondrial reactive oxygen species generation in blood cells is associated with disease severity and exercise intolerance in heart failure patients. Sci. Rep..

[25] Zhou B, Wang DD, Qiu Y (2020). Boosting NAD level suppresses inflammatory activation of PBMCs in heart failure. J. Clin. Invest..

