# Supplementary material for: Effects of metformin and donepezil on the prevention of doxorubicin-induced cardiotoxicity in breast cancer: a randomized controlled trial
Source: Sci Rep. 2023 Aug 7;13:12759. doi: 10.1038/s41598-023-40061-4 (PMC10406870; doi:10.1038/s41598-023-40061-4)
Supplement: Supplementary file 1 — Supplementary Table 1. [file 41598_2023_40061_MOESM1_ESM.pdf]

Supplement Table S1. Adverse events

Symptoms	Metformin (n=43)	Donepezil (n=52)	Placebo (n=48)	p-value
Dizziness	5 (11.63)	13 (25)	4 (8.33)	0.050
Nausea/vomiting	8 (18.6)	25 (48.08)	3 (6.25)	<0.001
Abdominal pain	2 (4.65)	2 (3.85)	0	0.34
Bloating	3 (6.98)	0	1 (2.08)	0.11
Diarrhea	11 (25.58)	7 (13.46)	3 (6.25)	0.032
Constipation	2 (4.65)	3 (5.77)	3 (6.25)	0.94
Nightmare	2 (4.65)	4 (7.69)	0	0.16
Insomnia	8 (18.6)	12 (23.08)	3 (6.25)	0.063
Palpitation	4 (9.3)	14 (26.92)	2 (4.17)	0.003
Fatigue	2 (4.65)	2 (3.85)	2 (4.17)	0.98
Chest pain	1 (2.33)	0	1 (2.08)	0.56
Pre-syncope	0	2 (3.85)	0	0.17
Dyspnea	1 (2.33)	0	1 (2.08)	0.56
Febrile neutropenia	0	1 (1.92)	1 (2.08)	0.65
